# Proposition of Belief and Practice Theory for Men Undergoing Infertility Treatment: A Hospital Based Study in Mumbai, India

**DOI:** 10.3389/fsoc.2020.00043

**Published:** 2020-07-28

**Authors:** Anshu Baranwal, Aparajita Chattopadhyay

**Affiliations:** International Institute for Population Sciences, Mumbai, India

**Keywords:** male infertility, treatment seeking behavior, process of treatment, infertility theory, Mumbai, India

## Abstract

The paper aims to understand the treatment seeking behavior and the experiences of men with male factor infertility. A cross-sectional study was conducted at consented hospitals/infertility centers in Mumbai, India in purview of the fact that men are not considered as important as a part of infertility treatment as women. An infertile man is defined here as one who is diagnosed with primary or secondary infertility, undergoing infertility treatment, irrespective of the fertility status of his wife. Primary data of 150 men undergoing infertility treatment from a variety of socioeconomic backgrounds was collected through semi-structured interviews. The initial effect of the infertility status led the men to feel depressed, guilty, shocked, and isolated. A large proportion of the respondents never discussed the problem with anyone except their wives. More than one third of the respondents consulted with Ayurvedic, Unani, Siddha, and Homeopathy (AYUSH) practitioners. Changes of doctors or clinics were more attributed to unsuccessful treatment cycles and success rate of other clinics than the referral by doctors. Destiny, bad luck, lifestyle, medical reasons, and late marriage are found as perceived causes of male infertility. Age above 40, younger age at marriage, marriage duration for 6 and more years, secondary infertility, self-employment, and higher income have significant association with longer time gap between marriage and initiation of infertility treatment. Based on study findings, we propose Belief and Practice theory where we elaborate the progression in treatment for male infertility. Men should be given due consideration in infertility treatment. They must be taken into consideration at an early stage of fertility evaluation due to the fact that minor problems of male infertility can be cured with modest medication. Proper Information Education and Communication (IEC) is essential for creating awareness in society on male infertility. Better counseling services during treatment and standardization of cost can help infertile men to manage treatment-related stress. Since infertility treatment is a time-consuming and exhaustive process, considering the timing for patient's income generating work, evening out patient department, and comprehensive knowledge dissemination at health centers can be improve male factor infertility treatment.

## Introduction

Infertility is a significant global health problem. One in six couples worldwide is affected by infertility. In more than half of the cases, the underlying problem of infertility is related to the male (Reuters, 2018). Yet research on male infertility treatment seeking is rare for two reasons: first, they are not considered as a primary client of infertility in spite of the evidence that there is an equally varied range of reasons for childlessness among men (Chattopadhyay and Mukherjee, 2015), and second, men themselves are apprehensive in talking about their infertility status. Traditionally, the female partner is assumed responsible for the failure to conceive. Pujari and Unisa (2014) in their study on childlessness in Andhra Pradesh, India, stated that most people acknowledge the role of female as well as the male factor in infertility but on a deeper mental make up it is implicitly a women's problem. The overpowered social structure of parenthood brings shame and disgrace to the infertile couple. Male infertility is often overlooked by doctors and the couple's infertility is most often perceived as a female health issue, when in fact the male partner may be having a male-specific problem (Chattopadhyay and Mukherjee, 2015). The reason for less emphasis is largely because of the fact that women receive the majority of medical and supportive attention from medical staff during examinations.

However, in half of the infertility cases, male reproductive capacity was found to be deficient (World Health Organization, 1987a,b). Men especially find it difficult to be at the center of infertility treatment as there is often no example in their social circle of such cases. Also, becoming a father is considered as an outcome of successful and powerful masculinity (Crawshaw, 2013). There has been a 20–30% rise in infertility cases, both in men and women in India, and male infertility has risen from 20% to around 50% in recent times (Diggikar, 2012). Male factor infertility is seen to have such social shame that it creates a large amount of negative social stress, confidentiality, and resentment (Peronace et al., 2007). Men have a 2–2.5 times less chance than women to see a doctor for a medical checkup, and therefore have fewer chances to be informed about the men's diagnosis and to go for infertility investigation (Eisenberg et al., 2013; Chandra et al., 2014). People who fail to have child are generally held responsible and stigmatized (Papreen et al., 2000; Dyer et al., 2004; Inhorn, 2004). Issues of male infertility arise mainly due to deficiency in semen, quantity, and quality of sperm (Cooper et al., 2010). The most common of these factors mentioned above are lower sperm count (oligospermia), poor motility of sperm (asthenospermia), and abnormal structure of sperms (teratospermia). There are many reasons for male infertility which affect the abnormal sperm count, such as childhood infections, hormonal disorders, genetic factors, and physical abnormalities. Extreme alcohol consumption in men has been associated with reduced reproductive abnormalities (Emanuele and Emanuele, 2001; Samal et al., 2012). Incidence of male infertility has increased due to various reasons such as environmental pollution, poor lifestyle, and stress (Mendiola et al., 2009; Hall and Burt, 2011).

Most of the research on male infertility is mainly conducted on the medical aspects. These studies were aimed at assessing the prevalence and etiology of male infertility (Irvin, 1998; Folkvord, 2005; Kumar and Singh, 2015; Sharma, 2017). More recent work suggests that men are now becoming more open about their desire to be fathers and about the choice to pursue fertility treatment (Mikkelsen et al., 2013). As treatments have advanced, there has been an enhanced role for males in assisted reproductive techniques. The advent of intra cytoplasmic sperm injection (ICSI) has brought a greater spotlight on the significance of the man's contribution to the *in vitro* fertilization (IVF) process (The ESHRE Capri Workshop Group, 2007; Merchant et al., 2011; Palermo et al., 2014; Sustar et al., 2019). Seeking medical help for infertility is not a simple process unlike other public health concerns such as tuberculosis or cancer. In this case, no individual seeks treatment to avoid immediate pain or death (White et al., 2006), but for having a child that renders long-term benefits. Seeking treatment depends upon the perceived cause of infertility, availability, affordability, and accessibility of various treatment options. Further, their own or others' experiences of infertility treatment determines their treatment-seeking behavior (Larsen, 2000). Family and couples use various traditional methods and religious practices to cure childlessness (Desai et al., 1992). Infertile people in developing countries seek treatment from biomedicine and more often from native health practitioners (Inhorn, 1994; Gerrits et al., 1999; Sundby, 2002; Barden-O'Fallon, 2005). Most of the couples combine their biomedical treatment with treatments based on traditional beliefs (Unisa, 1999; Mulgaonkar, 2001). Herbal drugs such as Ayurveda, Sidha, Unani, and Chinese traditional medicine are widely accepted to cure male infertility irrespective of not having scientific proof of their effectiveness (Chin et al., 2006).

Higher socioeconomic status is linked with greater use of infertility treatments with an affiliated increase in the cost. Higher income and education lead to increased utilization of health care through better awareness of health care options and higher utilization of advanced reproductive technologies (Smith et al., 2011). Family with more earnings have a choice to pay such services out of pocket. In contrast, households with lower incomes may limit the number and types of infertility treatments (Zegers-Hochschild et al., 2009). According to Indian Council of Medical Research [Indian Council for Medical Research (ICMR) and National Academy of Medical Sciences (NAMS), 2005], about 8% of infertile couples need serious medical intervention involving the use of advanced assisted reproductive technologies (ART) procedures such as IVF or ICSI. ICMR reported that there are an expected 250 IVF clinics in India at present. But the chance for successful ART treatment is <30% under best circumstances. IVF is not suitable for many couples because sperm count, motility, and morphology are of poor quality. Advanced alternate methods like ICSI are the largely established option for severe male factor infertility. Though ICSI is a revolution in treating male infertility, extensive use of this technique creates alarm about the transfer of genetic defects to upcoming generations [Indian Council for Medical Research (ICMR) and National Academy of Medical Sciences (NAMS), 2005]. There is little known about men's experiences of involuntary childlessness and how men with male factor infertility seek help. Male fertility is declining over past decades across the world (Auger and Jouannet, 1997; Benshushan et al., 1997; Feki et al., 2009; Huang et al., 2010; Jorgensen et al., 2012). Yet, literature on male infertility is minuscule in developing countries like India.

In the given context, this study evaluates men's reproductive health-seeking behavior and their experiences of involuntary childlessness. As the number of studies on male infertility is small in India where fertility is still considered to be a feminine issue of discussion, this research may help advance knowledge addressing infertility and its treatment.

## Methods

This is a cross-sectional study. Primary data was collected through semi-structured interviews of infertile men from hospitals/infertility clinics in Mumbai city. This study was carried out between July 2014 and December 2014. The steps adopted in data collection are given in Table 1.

**Table 1:** Procedure for data collection.

**Processes**	**Private hospitals**	**Infertility clinics**	**Public hospitals**
Health centers contacted	3	6	5
Procedure for permission	Permission from Ethics Review Board	Permission from the head of the clinic	Permission from Ethics Review Board
Agreement	Acceptance from 2 centers	Acceptance from 1 center	Rejected by 4 hospitals during initial conversation with the saying that no one from outside of the hospital is allowed to conduct the study. The fifth hospital had a long waiting time (1 year) to get clearance from the ethics review board.
Location	Center 1: North central region of Mumbai Center 2: South Mumbai	South Mumbai	
Facilities available	Center 1: Two gynecologists, one urologist, one embryologist, and other staff; Center 2: Two gynecologists, one embryologist, and other staff Both centers were equipped with full range of infertility treatment	Two gynecologists, one embryologist, and other staff equipped with full range of infertility treatment	Basic tests like semen analysis are done and only lower intensity treatments like IUI are provided. No advanced techniques are available.
Timing of data collection	8 a.m.−4 p.m.	6 p.m.−10 p.m.	Survey not conducted; however, few interactions with concerned authorities were made while seeking permission
Interview completed	First center: 68 Respondents Second center: 18 Respondents	64 respondents	

### Steps in Data Collection

Three established fertility centers in Mumbai, located in the north, central, and southern parts of the metropolis, had given consent to conduct the study out of 14 centers contacted by the authors. All three centers provided a complete range of infertility treatments including intrauterine insemination (IUI) with partner/donor sperm, surgery, and ICSI. The study sample included men of both primary and secondary infertility, at different stages of infertility treatment. The study was approved by the ethical approval board of the hospitals and the doctoral committee of the International Institute for Population Sciences, Mumbai, that functions under the aegis of the Ministry of Health and Family Welfare, Government of India. A semi-structured questionnaire was used. Open-ended questions were added to understand the feelings and experiences of men dealing with infertility.

Initially, the infertility consultant introduced the researcher to the patients by giving brief information about the study. Further, the researcher explained the study information and provided an invitation letter to the patient undergoing infertility treatment. The invitation letter mentioned the purpose and confidentiality of the study. Patients, if agreed, were requested to sign the consent form. In the consent form, it was mentioned that the information given by them would be kept strictly confidential and used for this study only. Further, they were assured that during the interview they could refuse to answer any question they did not want to answer or they could end the interview at any time. The study ensured the anonymity of the subject by replacing the respondent's names with unique identification numbers before statistical analysis. The hospital environment had no chance in influencing the responses as it was a closed room interaction with the respondent in absence of any other medical staff or relatives. We agree that a few respondents skipped questions or left the study in between for time constraints or personal reasons. Nevertheless, some respondents even said that they wanted to speak to somebody or share their feelings without being judged.

During the data collection, at the beginning of the conversation, some felt a little hesitant due to discussion on a topic that is sensitive and personal. Nonetheless, gradually they felt relaxed, took an interest, and provided most of the information. It was to the surprise of the interviewer that many discussed in length their difficulties related to infertility and its treatment, which they could not even share with the doctor or their family members. Some of the men showed interest in knowing the results and gave their contact details to convey the findings of the work upon completion.

### Theory and Conceptualization

Infertility is generally theorized based on scientific facts and psychological observations. These theories adopt the principles of description, forecast, and management of infertility (Covington and Burns, 2006). Olshansky (1987) proposed a psychoanalytical theory of identity for infertility. According to this theory, both males and females with infertility assume deficient identity by generating feelings of emptiness, despair, and disgrace. He further suggests that the person with infertility should incorporate it into their self so that he/she may be able to look beyond the deficient identity and move forward in life. Stress and coping theory, proposed by Taymor and Bresnick (1979), found that while both men and women experience the stigma of infertility, men seem to face more disgrace and inferiority no matter what infertility diagnosis is. Stage theory (Blenner, 1990) describes the experiences of couples from pre treatment to post infertility treatment. He proposed three stages of infertility treatment: (1) engagement, (2) immersion, and (3) disengagement. Kikendall (1994) proposed self-discrepancy theory. This theory explains that a woman with infertility experiences an identity crisis between her real identity as a woman and her role as a mother. Diamond and Kezur (1999) proposed phase theory. They divided the experience of a couple undergoing infertility treatment into five phases: (1) dawning, (2) mobilization, (3) immersion, (4) resolution, and (5) legacy, and discussed how couples feel the stress at each level from understanding the problem to go for other methods of family building like donor gametes, embryos, and adoption. Family systems theory (Daly and Kerry, 1999) looks into various factors affecting infertility as a genetic ailment, alternative options to expand the family, and impact of third-party transfer on families. This theory facilitates the counseling of the infertile couple.

Theories related to infertility mostly revolve around the psychological issues of infertile couples or the infertility treatment process, and most of these theories are based on developed countries. Infertility and treatment seeking is profoundly affected by stigma, social norms, and financial burden due to high cost of treatment, especially in developing countries like India.

**Figure 1:**
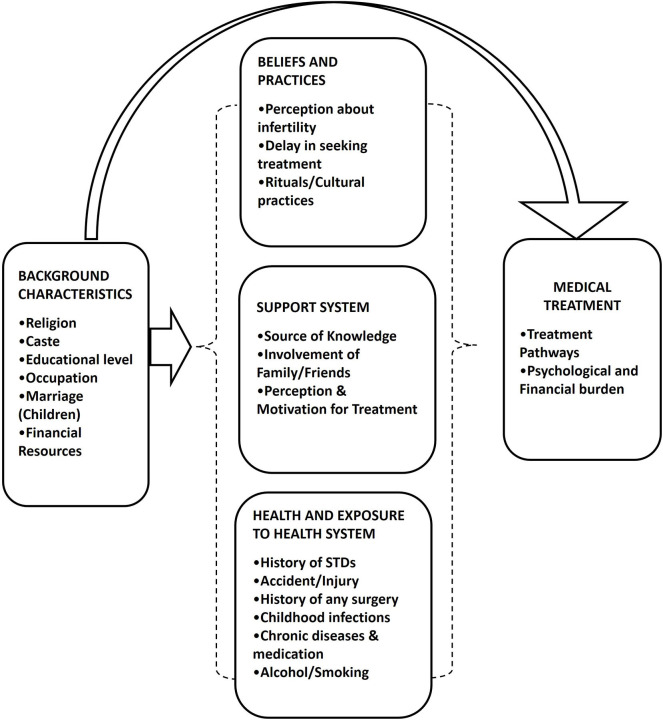
Treatment seeking framework for infertility.

While conceptualizing the research issue, we developed a treatment-seeking framework for infertility in our study (Figure 1). We referred the work proposed by Davis and Dearman (1987) and the framework for patient-centered fertility treatment proposed by Dancet et al. (2012). Davis in her framework describes the effect of the personal system, interpersonal system, and social system on infertility. Dencet et al. in their framework look at treatment through the patient's point of view and simultaneously consider four treatment dimensions: burden, effectiveness, safety, and costs of infertility treatment.

The framework (Figure 1) adopted in the present study shows the factors affecting infertility treatment seeking that depends mainly on the three components: first, the beliefs and practices; second, the perception about infertility and support system from family and society; and third, the health condition of a person and exposure to health systems. People's perception about infertility, acceptance, and reaction to it, and choice of treatment depends on existing beliefs and practices. Many times people use different ritual and cultural practices/cures as an option for infertility treatment instead of going for exhaustive medical treatments by authorized medical practitioners. Such practices are continued simultaneously with the medical treatment or when treatment cycles fail. Perceptions of society, their acceptance and support affect treatment seeking, as shame, stigma, and anti-masculinity notions are attached with infertility. Reaction, pressure, and support from family and friends influence the childless person as these factors interact in a close proximity. Medical history, exposure to health system environments, and previous experiences of medical treatment help to initiate the infertility treatment all are contextualized by socioeconomic status. During the treatment, people opt for different types of available treatments such as allopathic and AYUSH treatment, and sometimes they combine both types of treatment (Baranwal, 2018). Infertility and its treatment generate a lot of psychological pressure and stress to the treatment seekers. Cost plays a very important role in infertility treatment as these treatments lead to out-of-pocket expenditure.

The questionnaire was built based on the above framework and literature available on the treatment-seeking behavior of infertile people. A pretest was conducted in Mumbai among patients seeking treatment in clinics other than those selected for study. It took an average of 20–25 min to complete the questionnaire.

### Participants

This is an exploratory study. After getting ethical clearance, the researcher tried to get as many patients as possible from selected medical centers, within limitations of time and cost. An inclusion criterion was adopted to select the appropriate respondents for the study, i.e., male who is diagnosed with primary or secondary infertility and undergoing infertility treatment, irrespective of fertility status of wife. A total of 180 males diagnosed with infertility and undergoing treatment in selected hospitals/clinics were enlisted based on eligibility, availability, and ethical consent. Out of them, 162 patients gave consent and participated in the interview, and 150 patients completed the questionnaire.

### Measures

To ascertain the feasibility of the study, a pretest was carried out in a hospital, not enlisted for the actual survey during June 2014. After making the required changes as mandated by the pre-test, the questionnaire was made final.

The questionnaire had two sections. Section one covered the information about the socioeconomic status of the respondent, i.e., age, caste, religion, education, occupation, type of marriage, the composition of children (if any), information on health conditions such as medical history, infertility diagnosis, accident/injury, any surgery, medication, smoking, and alcohol use, major medical illness such as blood pressure, diabetes, trauma to testes, problem in erection, etc. In section two, questions related to their treatment-seeking behavior were covered. Beliefs and cultural practices were captured through realization and communication of the problem, religious practices, and perceived causes of infertility. To understand the perception and support system, questions on referral/recommendation for treatment, reason for seeking treatment, type of providers, etc., were included. Also, treatment-seeking pathway was captured through progression of allopathic and AYUSH treatment. There were some open-ended questions to understand their experiences and feelings. There were questions on the ways the patients fund their treatment and its implication on their financial status such as using savings, postponing other plans, etc.

Because it is a sensitive topic, we discussed many issues not mentioned in the interview schedule; we had a check list of topics, for capturing subtle emotions and stress, based on which we added verbatims.

### Data Analysis

Followed the conceptual framework as a path for data analysis to understand the treatment seeking behavior, the results obtained are discussed under four heads: (a) the mean time gap of treatment seeking between marriage, the realization of the problem, and the first or current infertility treatment for men having primary and secondary infertility; (b) the behavior related to infertility treatment such as initial reaction, communication regarding the infertility problem, religious practices to cure infertility problem, and perceived reported causes of infertility; (c) the process of infertility treatment such as referral for infertility treatment, type of providers, the number of providers visited during infertility treatment, and intensity of undergoing infertility treatment; and (d) treatment-seeking pathway of infertile men to different providers until the survey date. Further we looked into the determinants of the time gap between marriage and initiation of infertility treatment. Narratives from open-ended questions of the respondents regarding issues and problems of male infertility and treatment seeking were also added.

Descriptive analysis includes frequency distribution, means, percentages, and confidence intervals. The presentation method includes pie chart and graphical presentations. To understand the predictors of the time gap between marriage and initiation of infertility treatment, linear regression was applied.

## Results

### Primary Vis-a-Vis Secondary Infertility

About 77% of the respondents were dealing with primary infertility, while 23% were suffering from secondary infertility in the study population.

### Demographic and Socioeconomic Profile

Table 2 shows the proportion of infertile men seeking infertility treatment by their background characteristics. Age is a crucial characteristic; a large proportion of the infertile men (44%) belonged to young and productive age (30–34 years), while <30% of men were 40–49 years. The mean age of the patients was 35.4 years; more than 60% of the respondents completed graduation or above. It was interesting to observe that men undergoing infertility treatment had varied monthly income ranging from <20 thousand to more than 80 thousand INR.

**Table 2:** Percentage distribution of respondents by background characteristics.

**Characteristics**		**Percentage**
Age	20–29	8.0
	30–34	44.0
	35–39	28.0
	40–49	20.0
Type of family	Nuclear	50.7
	Joint	49.3
Caste	SC/ST	26.7
	OBC	24.7
	Others	48.7
Religion	Hindu	70.0
	Muslim	14.0
	Others	16.0
Education	1–8 years	10.0
	9–12 years	26.7
	Graduate	39.3
	Post graduate and above	24.0
Monthly income (Self)	<20,000	21.3
	20,000–39,999	30.7
	40,000–79,999	19.3
	≥80,000	28.7
Place of residence	Mumbai	90.7
	Not from Mumbai	9.3
Working profile	Govt. organization	18.7
	Private sector	53.3
	Self employed	16.0
	Others	10.0
	Not employed	2.0
Work shift	Up to 8 h	22.7
	9–12 h	60.0
	>12 h	17.3
*N*		**150**

### Lifestyle, History of Illness, and Diagnosis

One third of the respondents were either past or current smokers, while 51% were either past or current drinkers. It was observed that more than 50% of men suffered from major medical illnesses such as problems with erection, treated for sexually transmitted disease/tuberculosis/any surgery in their lifetime.

**Figure 2:**
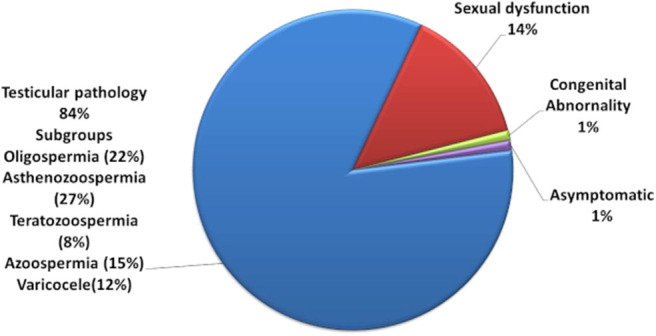
Infertility diagnosis of the respondents.

Out of the total, 72% of them were suffering from testicular pathology; 14% of the respondents had problems of sexual dysfunction (ejaculatory or erectile dysfunction), while 12% of the respondents were suffering from the problem of varicocele (Figure 2).

### Infertility Treatment and Treatment Seeking

**Figure 3:**
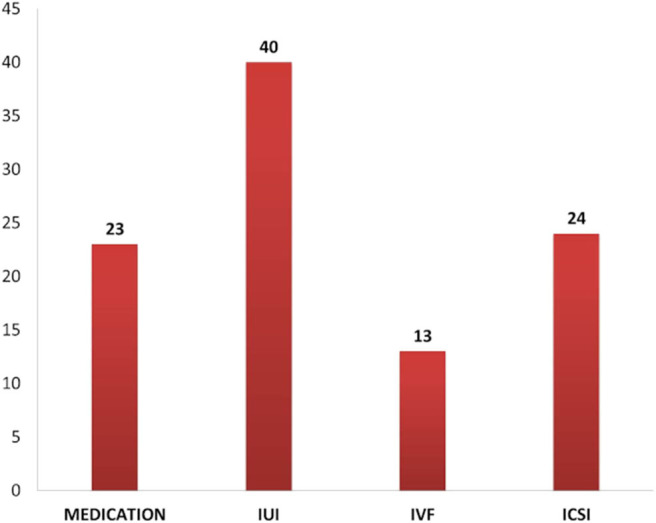
Infertility treatments of the respondents at the time of interview.

The problem of infertility has not been given its due attention as it is not a life-threatening condition. Patterns of treatment seeking depend on the couple's socioeconomic status, decision-making within the family, the level of information, and accessibility of treatment (Iyengar and Iyengar, 1999). Men undergoing infertility treatment may not represent the common population of men suffering from fertility issues as demographic, economic factors, and also the availability of a male consultant are influencing factors in the case of men seeking infertility treatment (Mehta et al., 2016). In the present study, it was found that almost 40% of the respondents were undergoing IVF/ICSI at the time of interview (Figure 3).

**Figure 4:**
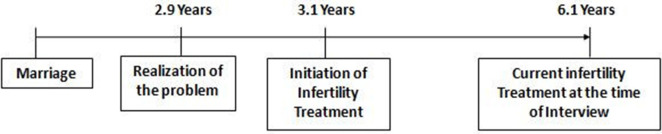
Mean time gap of treatment seeking (primary infertility).

**Figure 5:**
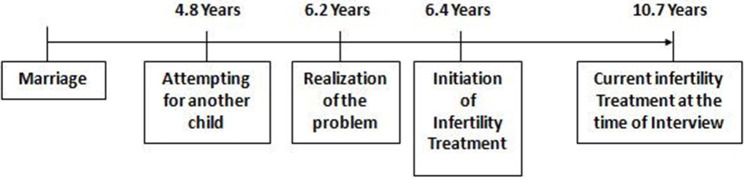
Mean time gap of treatment seeking (secondary infertility).

Figures 4, 5 show the mean time gap of treatment seeking. It is the time interval between marriage, the realization of the problem related to conception, and the initiation of infertility treatment. Out of 150 respondents, for 115 men who were suffering from primary infertility, the mean time gap between realizing the problem in conceiving and seeking initiation of infertility treatment was found as 0.2 years or 2.6 months. The mean time gap between marriage and the current infertility treatment at the time of the interview was found as 6.1 years.

Among 35 respondents who were suffering from secondary infertility, the meantime gap between realizing the problem in conceiving and seeking initiation of infertility treatment was found to be 0.2 years or 2.6 months, the same as in the case of primary infertility. The meantime gap between marriage and the current infertility treatment at the time of the interview was found as 10.7 years.

In the present study, we tried to understand the behavior related to infertility treatment (Table 3). To understand the initial reaction on infertility status, the respondents were asked, “*What was their first reaction to know that there is issue of fertility with them?*” Almost 30% of them said that they felt disappointed, and more than 25% of the respondents were very surprised or in shock to know their infertility status. Respondents also reported feeling guilty (24%) after knowing their status of infertility. Some of them also stated depression (14%), while few reported stress (6%), and 3% of them reported that they went into isolation after knowing the medical diagnosis.

**Table 3:** Behavioral characteristics of respondents by treatment seeking.

**Behavior**		**Percentage**
Initial Reaction on infertility status	Disappointment	27.0
	Very surprised/shock	26.0
	Feeling of guilty	24.0
	Depression/dejection	14.0
	Stress	6.0
	Isolation	3.0
	***N***	**150**
Communication of the problem (excluding wife)	Parents	37.0
	No one	32.0
	Brother	14.0
	In laws	13.0
	Friend	4.0
	***N***	**150**
Religious practices (multiple response)	Performing sacred rituals	48.0
	Wearing charms/stones	22.0
	Consulting faith healers/astrologers	16.0
	Visiting religious places	12.0
	Meditation	2.0
	**Response**	**82**
	***N***	**60**
Perceived reported causes (multiple response)	Life style factors	25.0
	Medical reasons	24.0
	Destiny/bad luck	26.0
	Late marriage	6.0
	Don't know	12.0
	**Response**	**93**
	***N***	**87**

A respondent aged 29 years suffering from azoospermia said,

“*I was aware that sperms can be weak, but I was in shock to know that I don't have sperms*.”

Another respondent, aged 34 years, commented,

“*When I came to know about my infertility status, I got so much stress that I could not even sleep for 15 days. I took sleeping pills for a long time to get sleep*.”

A respondents aged 30 years opined,

“*I felt guilty that I am not capable to give birth to a child. But this was the reality; I had to accept it anyway*.”

To know how they communicated about their infertility status in the family, the respondents were asked, “*To whom did they discuss their status in the family first?*” All the respondents unanimously reported that their wife was the first person with whom they discussed the matter. Out of the total respondents, 32% said that they never discussed the problem with anyone except their wives. Almost 40% of the respondents discussed the matter with their parents after discussion with their wives, while 31% of the respondents discussed the issue either with their brother, in-laws, or friend in addition to their wife.

To gather the information on decisions about infertility treatment, respondents were asked, “*Who in the family took the decision to opt for a medical treatment for infertility?*” In more than 21% of cases, respondents themselves took the decision for medical treatment, while in almost 70% cases both husband and wife together decided to opt for medical treatment; in 5% of cases the wife made the decision, while for the remaining 5% of cases parents and other family members decided to pursue medical treatment.

In order to understand the religious practices people perform to have a child, respondents were asked “*whether they ever visited any religious guru, faith healer or involve in any other religious/traditional practices?*” Out of 150 respondents, 60 respondents confirmed the use of some kind of religious practice for having a child. Out of these, performing sacred rituals (performing puja/hawan, observing fast) came out as the most preferred religious practice (48%), while meditation (2%) was opted as the least performed ritual. There were 22% of the instances of wearing charms (wearing tabeez, stone, ring, mala). Consulting faith healers or astrologers (visiting babas, showing kundli) and visiting religious places (temple, dargah, church) were found for 16 and 12%, respectively.

One of the respondents, aged 42 years, said,

“*We have done everything to solve this problem as suggested by others in the last five years. We visited all types of religious places, saw astrologers and worn stones as suggested by him, my wife observed fasts for me*.”

To grasp the perceived causes of childlessness, respondents were asked, “*what or who they think is the cause of not having a child?*” Out of 150 respondents, 87 respondents expressed their perceived cause of childlessness. Destiny or bad luck (God's wish, bad *karmas*, bad omen, *jadutona, najar lagna*, sins of last birth) was mentioned by 26%. Lifestyle factors (alcohol/smoking, work pressure, stress, pollution, no exercise, obesity, masturbation, and lack of interest in a sexual relationship) and medical reasons (illness, injury, surgery, genetic reasons) were usual causes of childlessness perceived by 49% of respondents. A few respondents said that they don't know (12%) why this complication is happening to them.

### Process of Infertility Treatment

Table 4 shows the process of infertility treatment like referral for infertility treatment, type of providers and the number of providers visited during infertility treatment, and intensity of undergoing infertility treatment in the present study.

**Table 4:** Characteristics of respondents by process of treatment seeking.

**Process of treatment**		**Percentage**
Referral Recommendation for infertility treatment (multiple response)	Self referred	23.5
	Wife	10.6
	Parents	17.1
	Friends	21.8
	Family doctor	15.5
	Previous doctor	11.6
	**Response**	**510**
	***N***	**150**
Decision for medical treatment	Both husband and wife	68.7
	Self	21.3
	Wife	5.3
	Parents	4
	Brother	0.7
	***N***	**150**
Reason for visiting doctor	Only willingness for child	60.7
	Too much family/social pressure	20.0
	Both willingness and family pressure	19.3
	***N***	**150**
Type of provider	Allopathic treatment	64.7
	Allopathic and AYUSH	35.3
	***N***	**150**
Number of doctor consulted	1–2	29.3
	3–4	46.7
	5 and above	24.0
	***N***	**150**
Type of infertility	Primary infertility	77.0
	Secondary infertility	23.0
	***N***	**150**
Diagnosis	Husband had a problem	60.0
	Both husband and wife had problem	38.7
	Asymptomatic	1.3
	***N***	**150**
Treatment category^*^	Lower intensity treatment (medication)	22.7
	Moderate intensity treatment (IUI)	40.0
	Higher intensity treatment (IVF/ICSI)	37.3
	***N***	**150**

* *Treatment category**-**Lower Intensity Treatment includes, respondents currently taking medication for infertility treatment Moderate intensity treatment includes, Intra Uterine Insemination as infertility treatment Higher Intensity treatment includes, in vitro fertilization/intra cytoplasmic sperm injection as infertility treatment*.

To understand the referral mechanism for infertility treatment, respondents were asked, “*who suggested them to consult the doctor for their problem*?” The study captured 510 responses for referral/recommendation to consult a doctor for an overall infertility treatment. Most of the referral for infertility treatment, i.e., almost 50%, was done either by friends or family. Around 25% of cases were found to be self-referred. While further probing on how these referrals were done, we found that the referrals were based on someone's successful infertility treatment outcome or how famous the fertility clinic/hospital is. Referral done by a family doctor and the previous doctor was found to be only 28%.

To be aware of the grounds for seeking medical help, respondents were asked, “*was there a social pressure for seeking treatment or was their personal need for the child that motivated them to seek treatment?*” Out of the total respondents, more than 60% of the respondents went for medical help as they were willing to have a child. Forty percent of the respondents sought medical assistance because of too much family/social pressure for having a child. Some of the respondents with secondary infertility told that they were seeking treatment because of the death of the previous child or in desire for a male child.

“*My only son of 17 years died in a road accident. We are now childless and want another child, but we are facing problem in conception. My brother referred me to this hospital for treatment*.”

To identify the type of treatment providers, respondents were asked “*what are the other places you have been to before coming to the current hospital for treatment?*” We found that almost two-thirds of the respondents (65%) sought only allopathic treatment, while more than one-third (35%) relied on both allopathic and AYUSH.

### Pattern of Treatment Seeking

**Figure 6:**
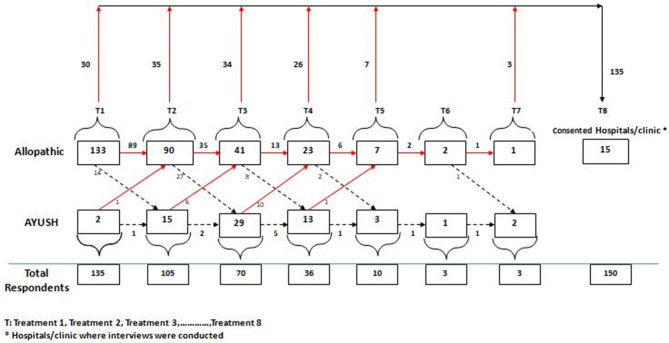
Treatment seeking pathway of the respondents.

To distinguish the respondents seeking treatment for infertility at various places, they were asked, “*As they felt the need to get infertility treatment, where did they visit for treatment (from the first contact to the last contact)?*” Figure 6 shows the treatment seeking pathway of the respondents for infertility treatment and the progression of males seeking infertility treatment at allopathic and AYUSH providers. It was found that out of 150 respondents, 15 respondents initiated their infertility treatment from the same hospital where the interview was held; 133 respondents began with allopathic treatment from somewhere else, while 2 respondents chose AYUSH as their starting point of treatment for infertility. Ultimately, these 135 respondents after seeking treatment at various places were undergoing treatment at the reference hospital at the time of the interview. Most of the patients chose the allopathic providers for infertility treatment, but there was an interchange of allopathic and AYUSH providers with the course of treatment. It was found that 65% of them took only allopathic treatment, while 35% of them took both allopathic and AYUSH treatments. No such respondent was found who took only AYUSH treatment. Further probing on the reasons to change the service providers found that such shift was either due to unsuccessful treatment outcome, because of the referral from previous doctor or family doctor, or due to referral from friends and family about a fertility center where the success rate was higher.

Table 5 shows the pattern of treatment seeking by type of providers. All men, irrespective of primary or secondary infertility, sought allopathic treatment, followed by 22% of men suffering from primary infertility, and almost 40% of men dealing with secondary infertility sought homeopathic treatment.

**Table 5:** Pattern of treatment seeking by type of providers.

Health care facility availed^*^	**Primary infertility**	**Secondary infertility**
	**Males (*N* = 115)**	**Males (*N* = 35)**
Allopathic	115 (100.0)	35 (100.0)
Homeopathy	25 (21.7)	13 (37.1)
Ayurvedic	13 (11.3)	8 (22.9)
Unani	2 (1.7)	5 (14.3)

* *Multiple responses*.

### Determinants of Time Gap Between Marriage and Initiation of Infertility Treatment

A linear regression analysis was applied to predict the determinants of the mean time gap between marriage and initiation of infertility treatment considering a set of predictor variables, i.e., background characteristics, marriage related characteristics, economic factors, health behavior, health condition, and treatment seeking behavior of the respondents (Table 6).

**Table 6:** Determinant of time gap between marriage and initiation of infertility treatment: linear regression.

**Variables**			**B**	
Background characteristics	Age	20–34^®^		
		35–39	0.757	(0.611)
		40–49	2.471^***^	(0.916)
	Religion	Hindu^®^		
		Others	−0.089	(0.514)
	Caste	OBC/SC/ST^®^		
		Others	0.726	(0.503)
	Type of family	Nuclear^®^		
		Joint	0.221	(0.464)
	Education	< Graduation^®^		
		Graduation and above	0.191	(0.669)
Marriage related characteristics	Duration of marriage	1–5 years^®^		
		6–10 years	0.728	(0.596)
		11 years and more	3.643^***^	(1.036)
	Age at marriage	Up to 28 years		
		More than 28 years	−1.280^**^	(0.558)
	Blood relatives having childlessness	Yes^®^		
		No	−0.095	(0.624)
Economic characteristics	Occupation	Government organization^®^		
		Private organization	0.450	(0.627)
		Self employed	1.224	(0.730)
	Income	<25,000^®^		
		25,000–50,000	2.010^***^	(0.697)
		>50,000	2.083^***^	(0.728)
	Working hours	Up to 8 h^®^		
		9–12 h	−0.030	(0.728)
		>12 h	−0.952	(0.836)
Health behavior	Smokers	Yes^®^		
		No	−0.285	(0.601)
	Drinkers	Yes^®^		
		No	−0.766	(0.494)
Health and treatment seeking	Major medical illness	Yes^®^		
		No	−0.947	(0.588)
	Diagnosis	Husband had problem^®^		
		Both husband and wife had problem	−0.144	(0.506)
	Type of infertility	Primary^®^		
		Secondary	1.318^**^	(0.608)
	Religious practices	Yes^®^		
		No	0.683	(0.483)
Constant		0.722		

® *Reference Category*,

** *p < 0.05*,

*** *p < 0.01; Standard errors are reported in parentheses*.

Age of 40–49 years [unstandardized coefficient (B) = 2.471, *p* < 0.01] significantly increases the onset of treatment with respect to 20–34-year-old men suffering from male factor infertility. Similar is the case for duration of marriage, i.e., 11 years and above marriage duration (B = 3.643, *p* < 0.01) was found to delay the treatment initiation compared to 1–5 years of marriage duration. The higher the age at marriage (above 28 years) (B = −1.280, *p* < 0.05), the lower the time gap between marriage and initiation of treatment.

Of the economic factors, income of Rs. 25000–50000 INR (B = 2.010, *p* < 0.01) and more (B= 2.083, *p* < 0.01) experience higher delay in treatment than those earning less than Rs. 25,000 INR. Those suffering from secondary infertility (B = 1.318, *p* < 0.05) had significantly more time gap between marriage and initiation of infertility treatment as compared to those with primary infertility.

Social factors like religion, caste, education, family, illness, and health behavior do not show any association with treatment delay.

The regression model explains 53% variability in explaining delay in treatment seeking [*F*_(22, 127)_ = 6.391; *p* = 0.000; *R*^2^ = 0.525].

### Financial Management of Infertility Treatment

To assess the cost of treatment, respondents were asked, “*how much cost did you pay approximately on your overall infertility treatment from the initiation of the treatment to the current treatment?*” The total average cost of infertility treatment, including all the previous and current treatment, was found to be more than Rs. 3 lakhs INR.

To understand how respondents managed to fund their treatment, they were asked, “*if their employer / medical insurance company provides any coverage for this treatment*.” Out of the total respondents, more than 14% of the respondents were having personal health insurance or covered with job's/company's health scheme. But all of them admitted that infertility treatment cost is not covered under health insurance or health scheme.

**Figure 7:**
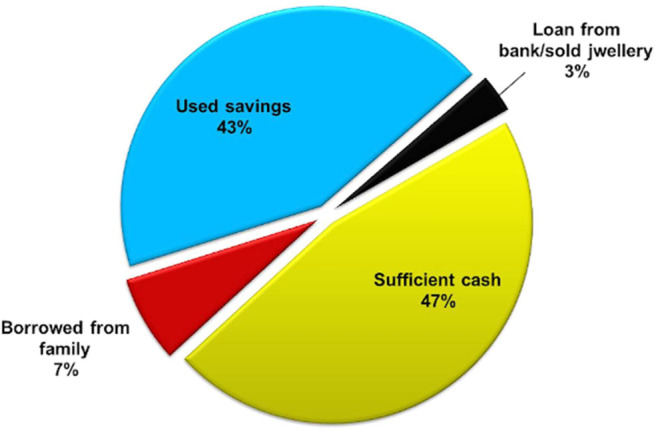
Source of money for treatment.

Out of 150 respondents, 129 patients responded to the question, “*how they managed to fund their treatment?*” Almost 50% of them told that they had sufficient amount of money for the infertility treatment, around 50% of the respondents used their savings for the treatment, 8% of the respondents said they had to borrow money from a family member/relative, while the remaining 2% of respondents took out a bank loan for their treatment (Figure 7).

We further asked, “*If the expenses of treatment has any impact on their future plans like savings/purchase of house etc*.” All the respondents unanimously accepted the very expensive and high-priced cost of infertility treatments.

Some of them considered the treatment costs as an obstacle for other life goals.

“*I have other life goals too. But because of these treatments, we are not able to save money for other important things in life. Our whole savings go into these treatments*.” (Respondent aged 30 years)

Few of the respondents expressed their willingness to stop treatment and adopt a child rather than spending on such costlier treatments.

“*We have taken treatment for Three years. Our Two IVFs got failed. We are undergoing the third IVF. We will not go to the next step of treatment if this IVF fails. Instead, we are thinking to adopt a child as we don't have another ten lakhs rupees to spend in such treatment where there is no surety of a successful outcome*.” (Respondent aged 35 years)

The expensive cost of treatment was found as one of the reasons for the late start of infertility treatment and also a reason for the time lag between the treatment cycles.

“*We have been trying for the baby for 13 years. We visited many doctors, but could not initiate treatment due to very expensive treatment options. We started treatment four years back only after we saved some money for the treatment*.” (Respondent aged 34 years)

“*We have already undergone two donor IVFs and have expended too much. Now if we have to repeat it, we will have to wait for at least one year to save some money for another IVF*.” (Respondent aged 36 years)

## Proposition of Belief and Practice Theory

Based on the results in our study, we propose Belief and Practice theory for men who are seeking treatment for infertility especially in the Indian context. Infertility treatment seeking is substantially different from other treatments due to stigma, secrecy, cost, and poor success rate associated with infertility treatment. Male infertility in the Indian context is treated much differently as fatherhood is seen as a symbol of masculinity. As per the proposed theory, seeking infertility treatment is not merely a treatment process of a disease but is influenced by complex social and psychological issues at an individual level and in society. This theory is applied to male infertility in various socio-psychological aspects, such as acknowledging and conceptualizing the problem, communicating with the family and society, the decision for treatment, and pursuing the treatment. It captures the process of infertility treatment seeking as well as the factors influencing the process of treatment seeking.

**Figure 8:**
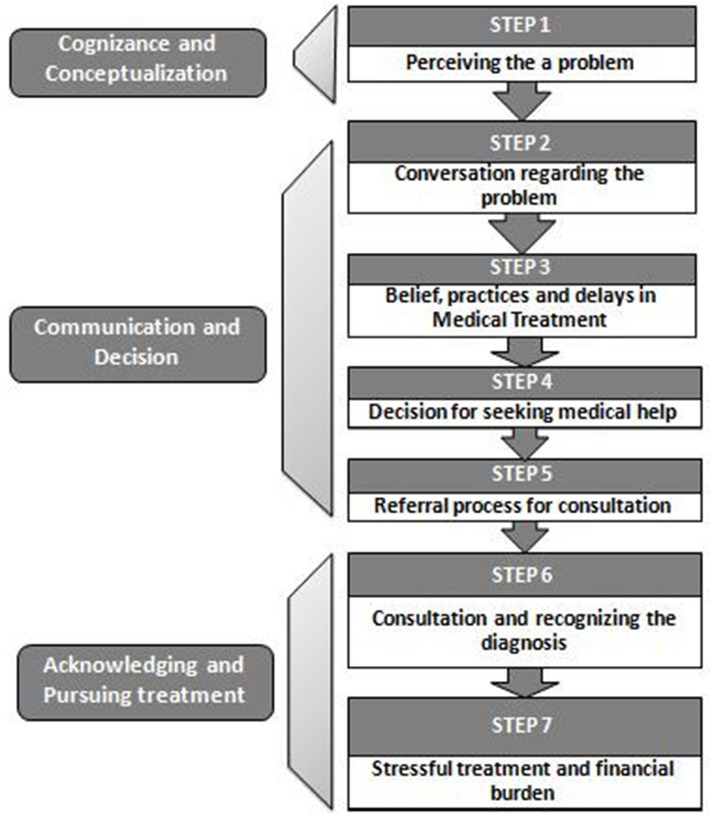
Belief and practice theory of treatment seeking.

This theory (Figure 8) is based on three concepts. (1) Cognizance and Conceptualization, (2) Communication and Decision, and (3) Acknowledging and Pursuing, as reflected in the proposed conceptual framework.

**(1) Cognizance and Conceptualization**

***Step 1: Perceiving the problem***

The first step of treatment seeking is initiated with the perception of the problem, when the couple start realizing the problem in conceiving. Perceiving the problem is not time-bound especially in India, where family and society start asking about “good news” (i.e., conception) after a few months of marriage. The problem in conceiving comes as a surprise as becoming a parent is considered as the most important phase of one's life in traditional society. Many couples initially do not take it seriously as they do not forsee problems related to conception. Even after repeated failure when they start seriously thinking about the issue, they comprehend that the problem is with the female partner due to lack of knowledge and masculine ideology. A respondent aged 31 years old said,

“*Initially we thought that we are facing problems in conceiving because of my wife's irregular periods. Therefore we kept on trying for long in the hope of conception someday*.”

**(2) Communication and Decision**

***Step 2: Conversation regarding the problem***

Secrecy and stigma attached to infertility affect the communication regarding the problem with the family members/relatives and friends. There is always a chance of getting uncomfortable remarks or nonsense advice. Men in the study are broadly divided into three groups based on their choice of the audience for the conversation on the matter. The first group of men never discussed the problem with anyone in their family (32%) except wives. The second group of men discussed the problem with their parents (37%) in addition to their wife, and the third group of men discussed the problem either with their brother, in-laws, or friend (31%) after their wife.

These interactions and reactions of family and friends played an important role in decision making for infertility treatment.

***Step 3: Belief, practices, and delays in medical treatment***

To overcome the problem of infertility, there are many practices adopted by the respondents and their families. Some of the respondents in our study stated that this problem is due to some black magic or evil spirit. Traditional and cultural practices were observed such as *puja/hawan, observing fast, wearing charms*, including consultation with faith healers or astrologers and visiting religious places (40%). All these practices were either advised by their family members or they decided to do so. These practices are done either before initiating the definitive infertility treatment or along with it. These practices lead to the delaying of medical treatment, where couples wait in the hope that these practices may resolve their infertility issues. But there is another group of men who reject these traditional practices, as observed in 60% of our respondents. They directly opted for consulting a gynecologist or family doctor. A respondent aged 38 years old said,

“*I don't believe in religious practices. But as my wife and family feel satisfied doing this, I also do for the sake of their hope and happiness*.”

Another respondent aged 40 years old said,

“*I am not a crazy man. I know these things never work. This is a biological problem and it can only be cured by medical treatments*.”

Belief and practices were used as a ray of hope and as a catalyst to the infertility treatment process.

***Step 4: Decision for seeking medical help***

Couple's strong desire as well as family pressure work to overcome the inertia for seeking infertility treatment. The decision to visit a medical facility or seek treatment is predominantly taken by couples, in a few cases by elders of the family. Based on reasons to seek medical assistance for infertility, men are divided into three groups in our study. The first group of men opted for medical help as there was excessive pressure from family (20%), while the second group went for medical help because they felt it as a personal need to have a child (61%). The third group of men decided to seek medical treatment due to both family pressure as well as a personal choice (19%).

***Step 5: Referral process for consultation***

A referral mechanism is found in the process of initial consultation with a doctor. In some cases, the gynecologist is prefered for consultation as the wife was already visiting for general health issues. Half of the cases in our study were referred either by family or friends (50%). These recommendations are done because either the consultant is a family doctor or someone in the family or friends received the successful infertility treatment from a particular consultant or clinic. In many cases, the male partner himself searched on the internet for the infertility centers or found advertisements and decided to meet a consultant, as observed for 24 cases in our study. Other major factors for clinic selection are distance of the clinic from home or office and the hours of the clinic as evening clinics.

- **(3) Acknowledging and Pursuing Treatment**

***Step 6: Consultation and recognizing the diagnosis***

During the initial days of consultation, tests such as scan and blood workup and HSG (Hysterosalpingogram) are done for the female partner, and if everything is found normal, they are given the advice to keep track of their menstrual cycle and keep trying for conception, or are given some medicine for ovulation and advised to try for conception on particular days of a menstrual cycle for at least 2–3 months. In the case of blocked fallopian tubes, laparoscopic surgery is advised. When this does not work, many couples stop taking advice from that consultant and start taking help from another practitioner due to mental stress, confusions, and quick remedies. Such switching of consultations is done again on someone's recommendation. It is observed that in few cases male's sperm count test is done after visiting the second or third doctor in the process of treatment seeking. But in most of the cases, tests are done for both male and female partners during the first consultation only. Reports confirming male infertility factor are shocking and uncomfortable news for men. For most of them, the point of accepting their infertility status is painful. They find themselves very surprised with disbelief and feel many emotions such as stress, isolation, and disappointment. Some of them also receive negative reactions from family members. A respondent aged 30 years in our study shared,

“*My brother asked me, ‘everyone in our family is having more than two kids. How come you are not able to do that?'*”

There are several views and perceptions among men regarding their infertility status. Most of them delve into questions like, Why me? What is wrong with me? How did this trait or abnormality seep into me? We found in our study that some considered the biological conditions such as higher age or late marriage as a cause of their fertility disorder, while some blamed their bad luck or past sins as one of the reasons for their sufferings; many considered poor lifestyles such as drinking and smoking, long working hours, or overuse of mobile phones as the reasons for their ailment.

A respondent aged 32 years said,

“*In my childhood, I always lived in a stressful family environment. My parents did not have a good relationship which made me very stressed and disturbed emotionally. I am suffering from this because of my previous traumatic life*.”

Some respondents said that the problem happened due to a lack of interest in sex or discontinuation of sex. A respondent aged 29 years told,

“*It may be because of my lack of interest in sex. I come home from the job very late at night. My job is very stressful. After coming back, I feel exhausted and don't feel making a sexual relationship*.”

A respondent aged 45 years old with secondary infertility quoted,

“*We did not make the sexual relationship for a long period, after the birth of my daughter. Whenever we did, we used the condom to avoid pregnancy. There was no direct contact between me and my wife for a long time. Now we want a child, but it is not happening because of previous use of precautions and long break*.”

Realization, acceptance, and way forward with new reality are seen as important characteristics of treatment seeking. In a few cases, it is also found that a female partner took the blame of having a fertility problem on herself to hide her male partner's infertility diagnosis, maybe because, in society, a female with reproductive issues is considered normal.

***Step 7: Stressful Treatment and financial burden***

Allopathic treatment is considered as a definitive treatment by them. Most of the men in the study started with allopathic treatment (98%) but shifted to Ayurvedic, Unani, Siddha, and Homeopathy (AYUSH) treatment to give it a try when they found that treatments were failing or not resulting in a success. Some of the respondents mentioned the lack of financial arrangements for switching over treatment from allopathic to AYUSH. There was always an interchange of allopathic and AYUSH treatment found with the course of infertility treatment. It was found that out of the total, 65% of them took only allopathic treatment, while 35% of them took both allopathic and AYUSH treatments. Many of the couples who were residing away from their family at their workplace were hiding their infertility treatment from their family. In a few cases, the male's in-laws were aware of the ongoing treatment while his parental family was not told about the treatment. Men also shared that they find it difficult to ask for leave from the office to get the treatment done as they don't want to share their infertility treatment to avoid unnecessary questions. A respondent aged 38 years old told,

“*I have not informed my family about my diagnosis and this treatment. Once we conceive, we will inform them*.”

At this step, couples are fully engrossed in the treatment. They start the treatment with full hope and assurance of successful treatment. As this is a cyclic process, if one treatment fails, one has to opt for advanced treatment options or repeat the treatment. Each time a treatment failed, it created a lot of anxiety and stress in couples. At each stage of treatment, there is a lot of pressure and fear about the result of the treatment.

Respondents expressed their anguish and disappointment at the failure of the treatment. A respondent aged 42 years, undergoing infertility treatment for the last 5 years, said,

“*After all four IUIs got failed, the doctor suggested us to opt for IVF. I was very hopeful until the first IVF was done. But when this also failed, my hopes shattered as this was the last option for the treatment*.”

A respondent aged 37 years, undergoing donor IUI/IVF, conveyed worry and anxiety of not being a biological father of the child.

“*We are using donor sperms as my sperms are not strong enough. I am going through lots of stress and extreme tension that the child will not belong to me*.”

The other unpleasant feelings men faced were guilt and helplessness about the treatment. A respondent aged 28 years said,

“*The problem is with me only, but my wife is the one who has to bear all the painful injections and procedures. I feel very sorry and very much worried about my wife's health*.”

Infertility treatments are very costly and take time. The cost of treatment acts as a barrier to get treated. In developing countries like India, infertility treatments do not come under insurance coverage, and advanced infertility treatments are available mostly in private hospitals and few tertiary care public hospitals. One has to bear a lot of financial burdens to carry on the treatment. All the respondents unanimously accepted the very expensive and high-priced infertility treatments. Many of them borrowed money from friends and family for treatment like IUI and IVF. A few respondents expressed their concerns about the success of these treatments and their willingness to stop treatment and adopt a child rather than spending on such costlier treatments.

A respondent aged 40 years said,

“*We have taken treatment for Three years. Our Two IVFs got failed. We are undergoing the third IVF. We will not go to the next step of treatment if this IVF fails. Instead, we are thinking to adopt a child as we don't have another ten lakhs rupees to spend on such treatment where there is no surety of successful outcome*.”

Infertility treatment is an elective treatment but it becomes important and necessary due to the social construct attached to it, that is, having your own baby is a must and without this life is incomplete. Infertility and its treatment is a complex situation subjected to uncertainty, hope, conflict of sharing, and secrecy. Individuals suffering from infertility struggle to cope interpersonally and in society during the process of treatment seeking. Costly treatment cycles and emotional and psychological cost affect the seekers terribly. Despite all costs involved, they opt for cycle after cycle of treatment with the frequent or infrequent change of doctors until they have the financial, mental, and physical capacity to seek the treatment.

## Discussion

The paper deals with the treatment-seeking behavior and experiences related to infertility of men who were seeking infertility treatment in hospitals/clinics in Mumbai. A number of 150 consented men having primary or secondary infertility from a variety of socioeconomic backgrounds participated in the study. Out of the total respondents, 77% suffered from primary infertility, while 23% were dealing with secondary infertility. The mean age of the respondents was found to be 35.4 years while 44% of the respondents belonged to young age (30–34 years). More than 70% of them were suffering from testicular pathology and almost 40% were undergoing IVF/ICSI at the time of the interview.

The meantime gap between marriage and the current infertility treatment at the time of the interview was 6.1 years for those with primary infertility and the meantime gap between marriage and current infertility treatment was found as 10.7 years for secondary infertility. We found that higher age, self-employment, longer duration of marriage, and type of infertility lengthens the time gap between marriage and initiation of infertility treatment.

Many participants were not aware of the infertility problem, and after knowing the problem it created mental pressure including anxiety, stress, and self-blame. Many of them felt depressed, some felt guilty, others shocked and isolated. Other studies also stated that men with infertility encountered many psychological and physical problems such as depression and erectile dysfunction (Sahin et al., 2017; Lotti and Maggi, 2018). They feel exhausted and miserable about their wedded life and relations due to incapacity to become a parent (Patel et al., 2018). Men generally don't express their emotions but take actions to cope with the situation or turn out to be a workaholic to avoid the stress of infertility (Pearson, 2019). In the present study, 12% of the respondents said that they don't know the reason for their childlessness. Another study conducted in Rwanda reported that more than 40% of men did not know the reason for infertility (Dhont et al., 2010). According to Pujari and Unisa, unawareness about reasons behind infertility is due to poor literacy and lack of media exposure of respondents (2014).

Almost one third of the respondents discussed the problem only with their wives. A retrospective cohort study done by Hammarberg et al. (2010) also reported that most of the infertile men discussed the problem with their intimate partner. In almost 40% of cases men consulted the doctor due to family pressure. Other studies also stated increase in anguish due to pressure created by family members for a child (Greil et al., 2013; Patel et al., 2018). It was found that many of the respondents in this study performed religious and cultural practices to cure infertility. Other studies also reported the use of cultural practices for the treatment of infertility. According to a study done by Desai et al. (1992), family and couples use various traditional methods and religious practices, including a visit to religious places, observing tantric rites, wearing charms, taking parts in rituals, and seeing astrologers. Another study stated that in developing countries, people avoid medical treatment for infertility due to shame, religious values, and social norms (Greil et al., 2013).

The present study found that respondents perceived destiny, bad luck, lifestyle factors, medical reasons, and late marriage as the causes of childlessness. In another community based study on childlessness (not specific to men) almost three fourth of the respondents attributed childlessness to reasons like fate, destiny, evil spirits, and God's will (Pujari and Unisa, 2014). A study done in the Middle East reported five factors that emerged repeatedly as the perceived cause of childlessness in men: heredity, illicit sex, civil war, stress, and pollution (Inhorn, 2012). Stress came out as the most reported cause of male childlessness (Bribiescas, 2001).

While looking into the treatment-seeking pathway of the respondents, it was found that initially, most of the respondents consulted allopathic providers for infertility treatment but there was an interchange between allopathic and AYUSH providers during the course of treatment. A study done on treatment-seeking behavior for infertility in Rwanda shows that only 5% of childless men had only consulted a traditional healer while the remaining had visited the formal medical sector (Dhont et al., 2010). Another study supports the use of homeopathic, ayurvedic, and use of religious practices for infertility treatment and also found treatment denial, dissatisfaction, and disappointment from assisted reproductive treatments (Vayena et al., 2002).

While asking about and the financial management of infertility treatment from the patient's perspective, we found that more than half of the respondents either used their savings or took loans for the treatment. Expensive infertility treatment caused financial distress in respondents and delayed their other life goals. Study on the cost of IVF treatment conducted in Kolkata, India, which included the only direct cost of IVF treatment, mentioned the average cost of a single IVF cycle vary between USD 1,300–USD 1,845, and patients found it difficult to manage the money for the treatment (Banerjee and Baranwal, 2020). Another study done in India found that monetary restrictions significantly increase anxiety in men and women undergoing infertility treatment (Patel et al., 2018). Some other studies also state that in developing countries, higher cost of treatment and stigma are the reasons for the dropout from higher end infertility treatments in many cases (Widge, 2002; Bharadwaj, 2003).

Infertility and its treatment are highly discriminatory due to the role played by the family, society, and also due to the financial implications/psychological burden related to infertility. Reaction from the family and society is very different and inconsiderate in the case of male infertility. The uncaring reaction, non-acceptance, and stigma attached to male infertility are due to the social construct and cultural norms of masculinity in the society. It becomes difficult for a person to seek treatment comfortably, to communicate and share his feelings, experiences, and problems with friends and family. “Male gender” itself is a critical barrier in male infertility treatment seeking.

## Conclusion

It is crucial to have a focus of reproductive health on men who are suffering from infertility as they silently deal with the problem in society with patriarchal set up. They are not given enough importance in the sense of treatment information and emotional well-being because men are considered less emotional and are supposed to support their female partners by typical masculinity norms. There is a need to bring men at par with women in infertility treatment. The proposed Belief and Practice theory highlights the role of family, society, and the cost of treatment that influence men seeking infertility treatment and thus an attempt to devise a male infertility treatment seeking theory in developing countries like India. This theory may help doctors, other medical staff, and researchers to understand the behavioral, societal, and emotional aspects of men undergoing infertility treatment for needed intervention on male infertility and its treatment.

Society, particularly men, should be made aware of causes of male factor infertility and its associated treatment. It can only happen when there is a proper awareness program or a media campaign to emphasize that male infertility is merely a disease. It may help infertile men to face the problem with dignity. During the treatment process, comprehensive information about investigation findings and potential treatment options should be provided in advance. The study suggests that it is necessary to provide good counseling services at every stage of infertility treatment to satisfy the queries and to control the anxiety of men who are going through mental stress. Improving psychological well being of men is a key to having a positive outcome. Higher end treatments such as IVF/ICSI are less time consuming but high-priced and thus restricted to only those who can afford it. To address the issue, the parts of infertility treatment processes that are very costly may come under the ambit of insurance coverage. Since infertility treatment is a time consuming, expensive, and exhaustive process, keeping the work day lost and cost for infertility treatment in mind, evening out patient department treating infertility at standardized costs are essential to address male factor infertility treatment.

## Limitations

Being a hospital based study, it was not possible to include childless men who were not taking any treatment for infertility. As this is a cross-sectional study, follow up was not possible to look into the complete journey of infertility/fertility. So, the potential areas of research could be a follow-up study to observe successful outcome, dropping out from treatment, or taking up other measures such as adoption and the impact of such decisions on childless men.

## Data Availability Statement

Due to the participant's confidentiality and privacy, the raw data generated in this study cannot be made available. The data presented in this article are in accordance with the ethical consent provided by the participants.

## Ethics Statement

This study was carried out in accordance with the recommendations of Institutional Review Board with written informed consent from all subjects. All subjects gave written informed consent in accordance with the Declaration of Helsinki. The protocol was approved by Institutional Review Board.

## Author Contributions

AB and AC: conceived and designed the study, data analysis, revision, and editing. AB: recruitment of respondents, data acquisition, and manuscript writing. All authors contributed to the article and approved the submitted version.

## Conflict of Interest

The authors declare that the research was conducted in the absence of any commercial or financial relationships that could be construed as a potential conflict of interest.
